# Fetal sex-specific epigenetic associations with prenatal maternal depressive symptoms

**DOI:** 10.1016/j.isci.2022.104860

**Published:** 2022-08-04

**Authors:** Michelle Z.L. Kee, Ai Ling Teh, Andrew Clappison, Irina Pokhvisneva, Julie L. MacIssac, David T.S. Lin, Katia E. Ramadori, Birit F.P. Broekman, Helen Chen, Mary Lourdes Daniel, Neerja Karnani, Michael S. Kobor, Peter D. Gluckman, Yap Seng Chong, Jonathan Y. Huang, Michael J. Meaney

**Affiliations:** 1 Translation Neuroscience, Singapore Institute for Clinical Sciences, A^∗^STAR, Singapore 117609, Singapore; 2 Bioinformatics, Singapore Institute for Clinical Sciences, A^∗^STAR, Singapore 117609, Singapore; 3 Department of Psychiatry, Douglas Mental Health University Institute, McGill University, Montreal, QC H4H 1R3, Canada; 4 Centre for Molecular Medicine and Therapeutics, BC Children’s Hospital Research Institute, Department of Medical Genetics, University of British Columbia, Vancouver, BC V5Z 4H4, Canada; 5 Department of Psychiatry, Amsterdam UMC and OLVG, VU University, 1007 Amsterdam, the Netherlands; 6 Department of Psychological Medicine (Mental Wellness Service), KK Women’s and Children’s Hospital, Singapore 229899, Singapore; 7 Department of Child Development, KK Women’s and Children’s Hospital, Singapore 229899, Singapore; 8 Centre for Human Evolution, Adaptation and Disease, Liggins Institute, University of Auckland, Auckland 1142, New Zealand; 9 Yong Loo Lin School of Medicine, National University of Singapore, Singapore 119228, Singapore; 10 Centre for Quantitative Medicine, Health Services and System Research Signature Research Programme, Duke-NUS Medical School, Singapore 169857, Singapore; 11 Department of Pediatrics, Yong Loo Lin School of Medicine, National University of Singapore, Singapore 119228, Singapore

**Keywords:** Biological sciences, Developmental biology, Neurogenetics, Developmental neuroscience

## Abstract

Prenatal maternal mental health is a global health challenge with poorly defined biological mechanisms. We used maternal blood samples collected during the second trimester from a Singaporean longitudinal birth cohort study to examine the association between inter-individual genome-wide DNA methylation and prenatal maternal depressive symptoms. We found that (1) the maternal methylome was significantly associated with prenatal maternal depressive symptoms *only* in mothers with a female fetus; and (2) this sex-dependent association was observed in a comparable, UK-based birth cohort study. Qualitative analyses showed fetal sex-specific differences in genomic features of depression-related CpGs and genes mapped from these CpGs in mothers with female fetuses implicated in a depression-associated WNT/β-catenin signaling pathway. These same genes also showed enriched expression in brain regions linked to major depressive disorder. We also found similar female-specific associations with fetal-facing placenta methylome. Our fetal sex-specific findings provide evidence for maternal-fetal interactions as a mechanism for intergenerational transmission.

## Highlights

• Maternal methylome significantly associates with prenatal depressive symptoms
• This association was *only* found in maternal methylome of mothers with a female fetus
• Genes from associated CpGs highly expressed in depression-linked brain regions
• Same female-specific association was found with fetal-facing placental methylome data

## Graphical abstract

**Figure:**
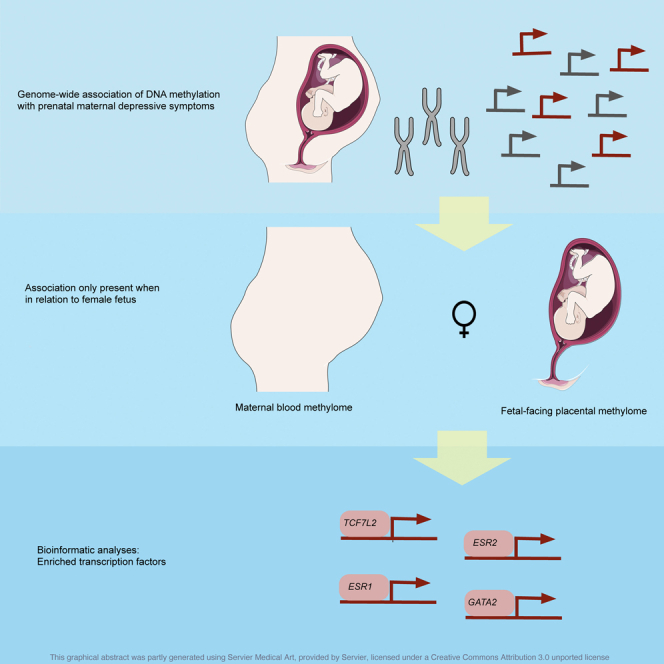

## Introduction

Perinatal maternal mental health is a major public health challenge, with significant long-term implications for the development and health of the offspring, including academic achievements and risk for psychopathology (Shen et al., 2016; Weissman et al., 2016). The economic toll of perinatal maternal mental health issues is estimated at billions of dollars annually (Bauer et al., 2014; Luca et al., 2020). At least one-third of the costs associated with maternal mental health problems are related to the adverse impact on the children. Recent studies also reveal that about 40% of pregnant mothers suffer from high, sub-clinical, and clinical levels of depressive symptoms (Meaney, 2018). Mothers with high, sub-clinical levels of depression show impairments in psychosocial function as severe as those with clinical levels of depression (e.g. Weinberg et al., 2001) with a significant impact on child neurodevelopment. An important consequence of maternal depression is a significantly increased risk for psychopathology in the offspring, including depression, internalizing and externalizing problems (see Goodman et al., 2011 for a systematic review).

An effective intervention for maternal symptoms of depression requires a thorough understanding of its underlying risk factors. Epidemiological studies identify psychosocial risk factors for poor maternal mental health including current life stressors and a lack of social support. However, the biological basis for inter-individual differences in maternal symptoms of depression is almost completely unknown. This gap derives, in part, from an earlier misconception surrounding dynamic variation in maternal mood over the perinatal period. The term “postpartum depression,” which remains in the medical lexicon, led to an understandable focus on the biological transitions that accompanied parturition, including those associated with pituitary-ovarian hormones. Detailed analyses of the large Avon Longitudinal Study of Parents and Children (ALSPAC) reveal slightly higher levels of depressive symptoms during pregnancy than in the postpartum period (e.g: Evans et al., 2001). These findings suggest that the biological origins for inter-individual symptom trajectories over the peripartum period are most appropriately examined during pregnancy and are, in most cases, unrelated to parturition. This conclusion is also consistent with subsequent longitudinal analyses in multiple cohort studies, which reveal that maternal symptoms of depression (or anxiety) are largely stable over the peripartum period, with only a rather small percentage of women showing a dynamic change at the time of delivery (Santos et al., 2017; Lim et al., 2019; Kee et al., 2021). Moreover, prenatal depressive symptoms are a better predictor of the risk for depression in the offspring than are those in the postnatal period (Pearson et al., 2013). These findings emphasize the importance of the prenatal period for studies examining the biological investigation of inter-individual variations in maternal symptoms of depression and possible mechanisms for risk of intergenerational transmission.

In this study, we examined the biological origins for variation in maternal symptoms of depression at mid-gestation in the Growing Up in Singapore Towards healthy Outcomes (GUSTO; Soh et al., 2014) cohort of pregnant women, using genome-wide epigenetic analyses focusing on DNA methylation. Whereas a substantial portion of the methylome is largely invariant within tissue type and across individuals, about 30% of CpG sites show the considerable inter-individual variation that primarily reflects genetic or gene x environment determination (Czamara et al., 2019; Hachiya et al., 2017; Teh et al., 2014). We thus reasoned that a genome-wide analysis of DNA methylation would inform on the complex underlying biological processes associated with variation in the quality of maternal mental health during pregnancy. We note that our analyses were not designed as an epigenome-wide association analysis to identify single epigenetic marks as candidate epigenetic “causal” mechanisms or biomarkers. We assumed that maternal symptoms of depression, as with those in the general population, are highly polygenetic, involve complex genetic and gene × environment interaction effects, and are thus not amendable to approach seeking singular causal events. Instead, we emphasized bioinformatic analyses that might inform on underlying biological processes through the analysis of genes bearing epigenetic modifications associated with maternal symptoms of depression.

Our primary objective was to examine inter-individual variation in DNA methylation profiles and associated biological processes as a function of prenatal maternal mental health. Interestingly, maternal prenatal health conditions, including asthma and blood pressure, vary as a function of fetal sex (Clifton and Murphy, 2004; DiPietro et al., 2011; Giesbrecht et al., 2015; Mitchell et al., 2017; Scott et al., 2009). Likewise, the child outcomes associated with prenatal maternal depression are highly sex-dependent (Paquin et al., 2020; Wen et al., 2017, and systematically reviewed in Meaney, 2018). We thus stratified the maternal methylome data as a function of fetal sex. As this study is, to our knowledge, the first extensive analysis of inter-individual variation in DNA methylation in relation to maternal mental health, we sought to examine if the fetal sex-dependent association between maternal methylome and maternal antenatal depressive symptoms was also observed in an independent dataset from the Avon Longitudinal Study of Parents and Children (ALSPAC) study that includes prenatal measures of depressive symptoms and genome-wide DNA methylation. We further stratified the fetal-side placental methylome as a function of fetal sex to elucidate the source whereby fetal sex could influence maternal methylome.

## Results

### Study characteristics

**Figure 1:**
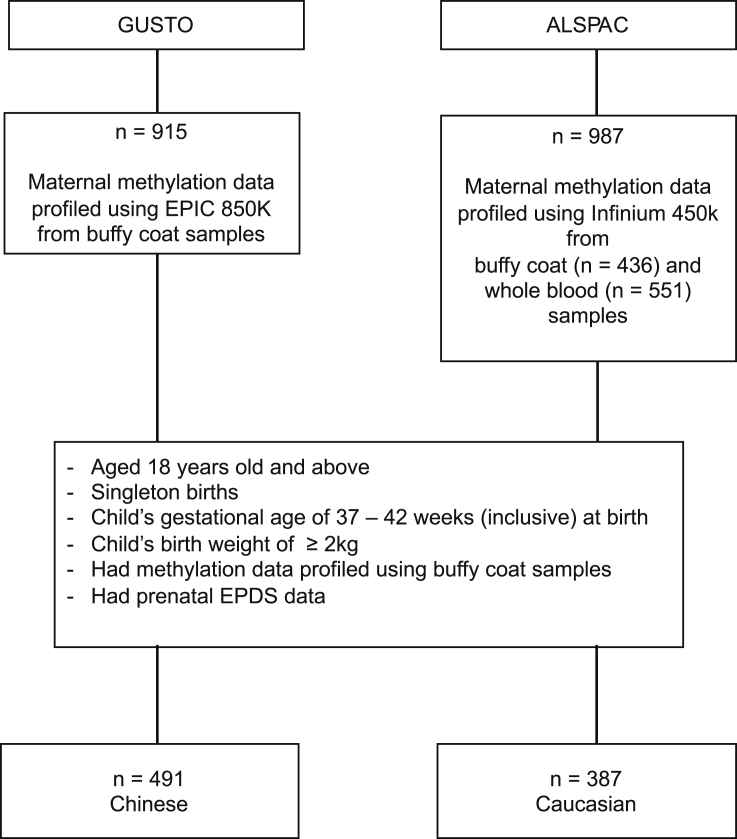
Flow chart showing sample selection from the ALSPAC and GUSTO cohorts ALSPAC, Avon Longitudinal Study of Parents and Children; GUSTO, Growing Up in Singapore Towards Healthy Outcomes; EPDS, Edinburgh Postnatal Depression Scale; Infinium 450k, Infinium HumanMethylation450 BeadChip; EPIC 850K, Infinium MethylationEPIC 850K BeadChip.

The sample selection from the GUSTO cohort is shown in Figure 1. Demographics of the GUSTO mothers of Chinese ethnicity who had both methylation and Edinburgh Postnatal Depression Scale (EPDS) data during their second trimester are summarized in Table 1. 47.7% of these 491 mothers bore female fetuses (*n* = 234). Mothers who were bearing either female or male fetuses were similar in EPDS scores (*t*(489) = 0.56, *p* = 0.58), as well as other demographics, which includes age (t(489) = 0.02, *p* = 0.99), marital status (χ^2^(2) = 0.71, *p* = 0.70), highest education levels attained (χ^2^(2) = 0.36, *p* = 0.84), monthly household income (χ^2^(3) = 1.49, *p* = 0.69; Table 1).

**Table 1:** Study characteristics

	GUSTO (mean ± SD)/n (%)^a^			ALSPAC (mean ± SD)/*n* (%)^a^		
	Female (*n* = 234)	Male (*n* = 257)	*p*	Female (*n* = 200)	Male (*n* = 187)	*p*
Maternal age at delivery (years)	32.3 ± 4.9	32.3 ± 4.5	0.99	29.5 ± 3.8	30.4 ± 4.5	0.03
Marital status			0.70			1
Married	221 (94.5)	244 (94.9)		172 (86.0)	162 (86.6)	
Single/Divorced/Separated/Widowed	8 (3.4)	10 (3.9)		28 (14.0)	25 (13.4)	
Missing information	5 (2.1)	<5 (<1.8)		0^b^	0^b^	
Maternal highest education			0.84			0.80
Secondary school and lower/CSE	52 (22.2)	63 (24.5)		9 (4.5)	10 (5.5)	
Pre-tertiary/Vocational/O level/A level	71 (30.3)	79 (30.7)		143 (71.5)	135 (72.2)	
Tertiary and above/Degree	108 (46.2)	114 (44.4)		44 (22.0)	37 (19.8)	
Missing information	<5 (<2.1)	<5 (<1.8)		<5 (<2.5)	5 (2.3)	
Monthly household income			0.69	NA	NA	
< S$ 2,000	20 (8.5)	23 (8.9)				
S$ 2,000–3,999	40 (17.1)	50 (19.5)				
S$ 4,000–5,999	62 (26.5)	57 (22.2)				
≥ S$6,000	93 (39.7)	108 (42.0)				
Missing information	19 (8.1)	19 (7.4)				
Crowding index	NA	NA				0.70
≤ 0.5				103 (51.1)	95 (50.8)	
>0.5–0.75				57 (28.5)	60 (32.1)	
>0.75–1				30 (15.0)	22 (11.8)	
>1				7 (3.5)	5 (2.7)	
NA				<5 (<2.5)	5 (2.7)	
EPDS scores during pregnancy	7.0 ± 4.4	6.8 ± 4.0	0.58	6.7 ± 4.6	6.6 ± 5.0	0.70
Pre-pregnancy BMI	21.3 ± 3.3	21.6 ± 3.3	0.41	NA	NA	
Child’s gestational age at delivery (weeks)	39.1 ± 1.0	39.1 ± 0.9	0.54	39.8 ± 1.2	39.6 ± 1.3	0.06
Child’s birth weight (kg)	3.1 ± 0.4	3.2 ± 0.4	2.00 × 10^−4^	3.4 ± 0.4	3.5 ± 0.5	0.02

*p*-values are based on Pearson’s chi-square tests for categorical variables and independent *t* tests for continuous variables.

a Percentages are rounded off to nearest 0.1%.

b This many include zero. NA = data not available.

### Fetal sex-dependent maternal methylome associations with prenatal maternal depressive symptoms

**Figure 2:**
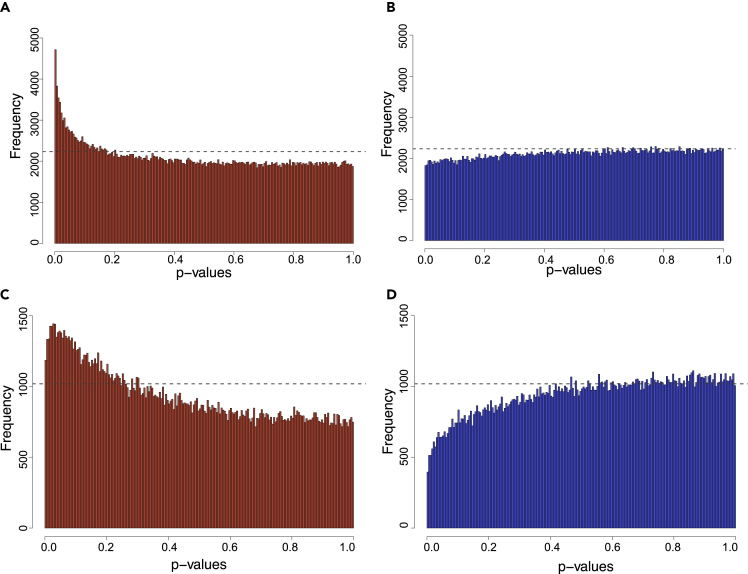
*p*-value distribution of maternal methylation associated with prenatal EPDS scores for mothers carrying either female babies (A, C) or male babies (B, D) Dashed lines represent the uniform distribution that was expected by chance. Top panels refer to data from GUSTO mothers, whereas the bottom panels refer to data from ALSPAC mothers.

DNA methylation profiling was performed using DNA extracted from maternal buffy coat samples and the Infinium MethylationEPIC 850K BeadChip (“EPIC 850K″) for the GUSTO cohort. We regressed variable CpGs (“vCpGs”; see STAR Methods for a detailed description of processing) from the maternal methylome onto prenatal maternal depressive symptoms using the scores from the EPDS. These analyses were further stratified by the sex of the fetus. We observed a characteristic non-random *p-*value range of EPDS-associated vCpGs skewed toward the low end for mothers bearing female fetuses (Figure 2A; Kolmogorov-Smirnov (KS) test *p* < 0.0001). In contrast, the p-value distribution for EPDS-associated vCpGs for mothers bearing male fetuses was no different from that expected by chance (Figure 2B; KS test *p* > 0.99). As the study was not designed as an epigenome-wide association study (EWAS) to identify single epigenetic marks, we identified vCpGs associated with prenatal maternal depressive symptoms at a nominal *p*-value < 0.005 as “EPDS-vCpGs,” instead of using the standard EWAS threshold. Mothers bearing female fetuses had 4,716 EPDS-vCpGs (1.1% of total vCpGs), ∼2.6 times more EPDS-vCpGs than peers bearing male fetuses (1825 EPDS-vCpGs; 0.4% of total vCpGs).

We proceeded to examine if similar fetal sex-dependent *p-*value distributions were observed in the ALSPAC cohort. DNA methylation profiling in the ALSPAC cohort was performed before the advent of the EPIC array on maternal buffy coat samples using the Infinium HumanMethylation450 BeadChip (“Infinium 450K”). The ALSPAC cohort is a community sample from the United Kingdom and was found suitable as both EPDS and maternal methylation from blood samples were obtained during pregnancy and postnatally (see STAR Methods). The cohorts also displayed a similar percentage of women exhibiting clinical levels of prenatal depressive symptoms, defined by an EPDS score of ≥15 (GUSTO: 4.7%, *n* = 23 out of 491; ALSPAC: ∼ 6.5%, *n* = 25 out of 387). However, some differences remained between the two cohorts. First, the differences in terms of demographics between the two cohorts are found in Table 2. Second, the EPDS was obtained at somewhat different timepoints during pregnancy for GUSTO and ALSPAC mothers; GUSTO samples were collected in the late second trimester, whereas samples for ALSPAC were collected mid-third trimester. Next, maternal methylome was profiled using EPIC 850K array in the GUSTO cohort, whereas maternal methylome was profiled using Infinium 450K array. Notwithstanding these differences, the ALSPAC cohort does provide a cohort to determine if our novel finding of fetal sex specificity was observed.

**Table 2:** Top 15 biological pathways of genes mapped from EPDS-vCpGs of mothers with female fetuses

No.	Biological pathways		*p*	FDR
1	Development	Positive regulation of WNT/Beta-catenin signaling at the receptor level	4.73E-11	6.80E-08
2	Main genetic and epigenetic alterations in lung cancer		1.26E-08	9.10E-06
3	Signal transduction	Adenosine A1 receptor signaling pathway	3.88E-08	1.78E-05
4	Putative role of Estrogen receptor and Androgen receptor signaling in the progression of lung cancer		6.41E-08	1.78E-05
5	Nociception	Nociceptin receptor signaling	7.42E-08	1.78E-05
6	Gamma-secretase proteolytic targets		7.42E-08	1.78E-05
7	Signal transduction	Cyclic AMP signaling	9.35E-08	1.92E-05
8	Development	Thromboxane A2 signaling pathway	1.71E-07	3.08E-05
9	Signal transduction	PKA signaling	2.38E-07	3.80E-05
10	Transcription targets of Androgen receptor involved in Prostate Cancer		4.39E-07	6.33E-05
11	Epigenetic alterations in ovarian cancer		1.38E-06	1.70E-04
12	Gamma-Secretase regulation of neuronal cell development and function		1.41E-06	1.70E-04
13	Signal transduction	Intracellular calcium increase	1.67E-06	1.85E-04
14	Signal transduction	WNT/Beta-catenin signaling in tissue homeostasis	2.76E-06	2.84E-04
15	Signal transduction	Angiotensin II/ AGTR1 signaling via p38, ERK and PI3K	3.32E-06	3.18E-04

See also Table S2 for top 30 biological pathways.

For a systematic methylome comparison, we extracted common vCpGs found in both cohorts and that were then associated with prenatal maternal depressive symptoms (*n* = 187,935). Similar to the findings from the GUSTO cohort, there were more EPDS-vCpGs with low *p-*values (*p* < 0.005) only in ALSPAC mothers bearing female fetuses (Figure 2C; KS test *p* < 0.0001). This finding is in contrast with the maternal methylome in samples of those mothers with male fetuses, which were all at the level expected by chance (Figure 2D; KS test *p* > 0.99). This fetal sex-dependent association was only observed in vCpGs. Non-variable CpGs showed no association with prenatal depressive symptoms (Figure S1). These analyses were repeated to identify EPDS-vCpGs at a nominal *p-*value threshold of <0.001. We observed a similar female-specific EPDS-associated vCpGs enriched at a low *p-*value range (Figure S2). The replication is thus not unique to a specific *p*-value. These analyses of the ALSPAC data show similar fetal sex-specific profiles based on the associations of DNA methylation and maternal depressive symptoms in the prenatal period.

### Fetal sex-specific genomic distributions of EPDS-vCpGs

**Figure 3:**
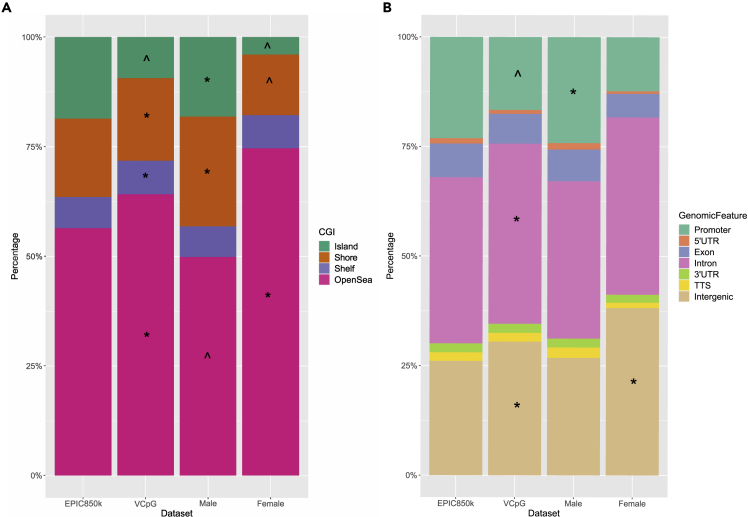
Genomic distributions of vCpGs CpG sites (A) and genomic region distributions (B) of vCpGs were compared with all CpGs present in EPIC 850K as reference, whereas EPDS-vCpGs in male and female were compared to vCpGs as reference. Bar plots showing genomic regions distribution. ∗ represents regions that were significantly enriched, whereas ˆ represents regions that were significantly depleted (both *p* < 0.05). See also Table S1.

We focused our subsequent analyses on the EPIC 850K methylation data provided by the GUSTO cohort, as the EPIC 850K array almost doubles the content of the Infinium 450K and provides improved coverage of the genome including broader information on genomic location. In contrast, the 450K array is heavily focused on CpG islands. We observed that the vCpGs, relative to the EPIC 850K reference CpGs, were significantly enriched (*p* < 0.05) in the open sea, intergenic and intronic regions, and significantly depleted (*p* < 0.05) in CpG islands and promoter regions (Figure 3).

We further examined whether the fetal sex-specific maternal EPDS-vCpGs showed distinct genomic features relative to the total vCpG probe distribution (Figure 3 and Table S1). We found that EPDS-vCpGs of mothers bearing female fetuses were enriched in the open sea (*p* = 2.31 × 10^−54^) and intergenic regions (*p* = 1.04 × 10^−28^). Conversely, EPDS-vCpGs of mothers bearing male fetuses were enriched in CpG islands (*p* = 4.28 × 10^−31^), shores (*p* = 4.07 × 10^−11^), 5′ UTR (*p* = 1.68 × 10^−2^) and promoter regions instead (*p* = 2.20 × 10^−13^). Relative to vCpG distributions, these regions were significantly depleted in EPDS-vCpGs of mothers bearing female fetuses. These findings reveal striking fetal sex-specific, regional differences in maternal methylome associated with prenatal depressive symptoms.

### Genes mapped from EPDS-vCpGs in mothers carrying female fetuses found in biological processes and brain regions implicated in major depressive disorder

We next explored the potential biological functions of EPDS-vCpGs in mothers bearing female fetuses and queried if genes mapped from these CpGs were enriched for particular biological pathways. The 2,417 unique genes mapped from 4,716 female fetus-associated maternal EPDS-vCpGs were most significantly enriched for pathways related to signal transduction and developmental pathways (see Table 2 for the top 15 pathways and Table S2 for the complete table for EPDS-vCpGs). Interestingly, the top pathway mapped from genes expressed in female fetus-associated maternal EPDS-vCpGs was the “positive regulation of WNT/β-catenin signaling at the receptor level” (*p* = 4.73 × 10^−11^; FDR *p* = 6.80 × 10^−8^). As noted below (see Discussion), this pathway has been closely linked to major depressive disorder (MDD). The EPDS-vCpGs were also enriched in genes regulated by several transcription factors. These include genes involved in the Wnt signaling pathway (e.g: *TCF7L2*), steroid hormone receptors (e.g. estrogen receptor alpha (*ESR1*), estrogen receptor beta (*ESR2*), and androgen receptor), and more notably, *GATA2*, a pioneer transcription factor (Table S3).

**Figure 4:**
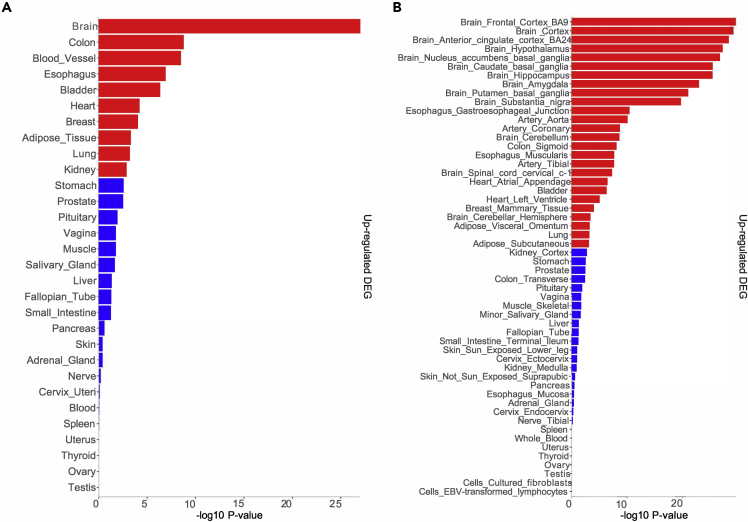
Tissue-specific expression of genes mapped from female fetus-associated EPDS-vCpGs relative to vCpGs from maternal blood methylome (A) Genes mapped from female fetus-associated EPDS-vCpGs from maternal blood methylome were highly expressed in the brain, and its specific regions (B). Significant enrichment at Bonferroni-corrected *p* ≤ 0.05 are in red, whereas *p* > 0.05 are in blue.

We used a web-tool FUMA (https://fuma.ctglab.nl/) that incorporates biological data repositories to further evaluate the roles of genes mapped from female fetus-associated EPDS-vCpGs. Interestingly, these sites mapped to genes that were also found in existing genome-wide association studies for psychiatric disorders including MDD (FDR *p* = 3.37 × 10^−4^) as well as schizophrenia (FDR *p* = 1.11 × 10^−5^) and psychosocial problems (e.g.: general risk tolerance; FDR *p* = 6.28 × 10^−10^). These genes mapped from female fetus-associated EPDS-vCpGs, relative to the genes mapped from vCpGs, were also found to be significantly differentially up-regulated in the brain compared with other tissues (Figure 4A). More specifically, these genes were found to be enriched in expression in brain regions including the frontal cortex Brodmann area 9 (BA9), anterior cingulate cortex Brodmann area 24 (BA24), basal ganglia, nucleus accumbens, hippocampus, and amygdala (Figure 4B).

### Similar fetal sex-dependent associations with prenatal maternal depressive symptoms using fetal-side facing placental methylome

**Figure 5:**
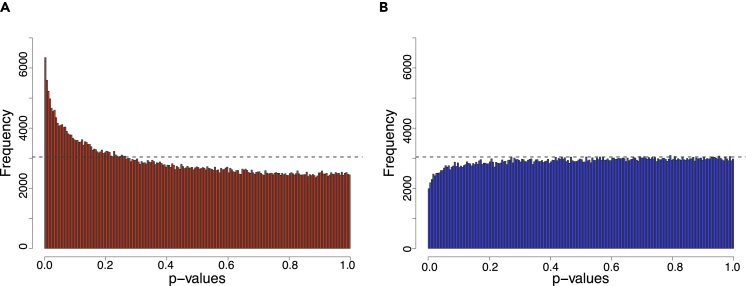
*p*-value distribution of fetal-facing placenta methylation associated with prenatal EPDS scores for female (A) or male fetus (B) Dashed lines represent the uniform distribution that was expected by chance.

We next focused on determining the source of the sex-dependent fetal response to antenatal maternal depressive symptoms. The placenta serves as the biological interface between the mother and the fetus. Hence, we examined the association between maternal depressive symptoms and the methylome of the fetal-facing placenta as a function of fetal sex. The fetus-facing placenta DNA methylation from the available GUSTO placental samples was profiled using the EPIC 850K (*n* = 125; 49.6% female fetal-facing; see STAR Methods for detailed sample description and analyses). Remarkably, the fetal-facing placenta methylome from female but not male fetuses revealed a characteristic non-random enrichment of a low *p-*value range of EPDS-vCpGs (Figure 5A; KS test *p* < 0.0001). The *p-*value distribution for EPDS-vCpGs for male fetal-facing placental methylome was no different from that expected by chance (Figure 5B; KS test *p* > 0.99). Non-variable CpGs showed no association with prenatal depressive symptoms (Figure S3).

The 3,619 unique genes mapped from 6,345 female fetal-side specific placental EPDS-vCpGs were further examined for biological pathway enrichment analyses. Interestingly, these genes were enriched in pathways fundamental for fetal development – specifically, neuronal signaling (e.g: *ACM1/3/5* signaling in the brain), neurite outgrowth (e.g: *RAP1A*), and axon guidance signaling (e.g: *Netrin-1*, *ROBO2/3/4* signaling pathways; see Table 3 for top 15 pathways, Table S4 for complete list). Interestingly, these female-specific fetal-facing placenta EPDS-vCpGs were also enriched for genes regulated by transcription factors as shown before in maternal samples, specifically *TCF7L2, ESR2*, and *GATA2* (Table S5). Taken together, these findings reveal that the fetal sex-specific associations with prenatal maternal depressive symptoms were present in both maternal blood and fetal-facing placental methylome. Hence, the placenta is a likely source of the fetal response to maternal depressive symptoms.

**Table 3:** Top 15 biological pathways of genes mapped from EPDS-vCpGs from female fetus placenta tissue (fetal-facing)

No.	Biological pathways		*p*	FDR
1	Development	NCAM1-mediated neurite outgrowth, synapse assembly, and neuronal survival	1.11E-06	1.656E-03
2	G-protein signaling	Rac1 activation	3.53E-06	2.295E-03
3	Neurophysiological process	ACM1, ACM3, and ACM5 signaling in the brain	4.63E-06	2.295E-03
4	Development	PIP3 signaling in cardiac myocytes	7.74E-06	2.877E-03
5	Protein folding and maturation	Insulin processing	1.26E-05	3.735E-03
6	Chemotaxis	SDF-1/ CXCR4-induced chemotaxis of immune cells	1.65E-05	4.090E-03
7	Neurophysiological process	Netrin-1 in regulation of axon guidance	3.58E-05	7.612E-03
8	Cytoskeleton remodeling	Regulation of actin cytoskeleton nucleation and polymerization by Rho GTPases	4.55E-05	8.028E-03
9	Immune response	Immunological synapse formation	5.08E-05	8.028E-03
10	Signal transduction	Cyclic AMP signaling	5.40E-05	8.028E-03
11	G-protein signaling	Rap1A regulation pathway	7.97E-05	1.077E-02
12	Transcription	CREB signaling pathway	1.07E-04	1.305E-02
13	Regulation of metabolism	GLP-1-induced insulin secretion	1.14E-04	1.305E-02
14	Development	ROBO2, ROBO3 and ROBO4 signaling pathways	1.42E-04	1.504E-02
15	Development	Role of HDAC and calcium/calmodulin-dependent kinase (CaMK) in control of skeletal myogenesis	1.70E-04	1.683E-02

See also Table S4 for top 30 biological pathways.

## Discussion

Our analyses reveal an association between antenatal maternal symptoms of depression and variation in genome-wide DNA methylome that is strikingly dependent upon the sex of the fetus. In both the GUSTO and ALSPAC cohorts the fetal sex-dependent effect was apparent in the *p-*value distribution for the association between DNA methylation and prenatal EPDS scores for mothers bearing female vs male fetuses (Figure 2). The association between maternal depressive symptoms and DNA methylation profiles was found during the prenatal period, which implicates fetomaternal signaling processes. A qualitative analysis of the genomic distribution of vCpGs significantly associated with maternal depressive symptom scores also reveals fetal sex-dependency. Relative to the EPIC 850K reference map, vCpGs in maternal blood associated with EPDS scores bearing female but not male fetuses were over-represented in intergenic/open sea regions and under-represented in the promoter regions within CpG islands (Figure 3). The fetal sex-dependent results specific to the females were also observed in the associations between fetal-facing placental methylome with maternal depressive symptoms. Finally, our informatic analyses link vCpGs associated with maternal depressive symptoms to genes, biological processes, and brain regions implicated in MDD.

We note there was no difference in the antenatal maternal EPDS scores as a function of fetal sex. This finding suggests that, instead, the results reflect a more direct effect of fetal sex. There are well-established, sex-dependent effects of antenatal maternal conditions on offspring health outcomes. The most commonly reported instances reflect a greater impact of a range of maternal conditions on male fetuses, especially for later neurodevelopmental outcomes including ADHD, autism, and schizophrenia (Bronson and Bale, 2016).

In contrast, maternal asthma or pre-eclampsia associates with normal growth trajectories of the male fetus but growth reduction in the female fetus (Murphy et al., 2003; Stark et al., 2009a). These outcomes are related to fetal sex-specific alterations in maternal physiology (Clifton and Murphy, 2004). These examples of sex-specific fetal outcomes to maternal health conditions are reflected in placental biology (Bronson and Bale, 2016; Scott et al., 2009). The fetal sex effect associated with maternal asthma is apparent in placental pro-inflammatory responses. Expression of the cytokines TNF-α, IL-1β, IL-6, IL-5, and IL-8 were increased in placentae of female, but not male fetus, in pregnancies complicated by asthma (Scott et al., 2009). Conversely, healthy women with male fetuses had higher IL-1β and plasma nitric oxide levels in early pregnancy, compared with their counterparts bearing female fetuses (Ramiro-Cortijo et al., 2020). Women carrying females also had higher levels of TNF-α in early pregnancy, IL-1β in mid-late pregnancy, and IL-6 throughout pregnancy (Mitchell et al., 2017). As noted above, maternal asthma is associated with a significant reduction in birth weight in female but not male neonates (Murphy et al., 2003). These alterations in growth in female fetuses were associated with both increased circulating concentrations of cortisol and greater glucocorticoid sensitivity as well as decreased placental cortisol metabolism by the protective barrier enzyme, 11β-hydroxysteroid dehydrogenase 2 (11β-HSD2) (Clifton and Murphy, 2004; Murphy et al., 2003; Stark et al., 2009b). Elevated exposure to glucocorticoids produces fetal growth retardation (Meaney et al., 2007). These findings are consistent with the positioning of the placenta as the interface between maternal health and fetal biology (Bronson and Bale, 2016).

Our findings suggest sex differences in fetal responses to the maternal depressive symptoms that, in turn, are reflected in maternal blood DNA methylation. The offspring of mothers with increased depressive symptoms, particularly during the prenatal period, have a higher risk for depression (Pearson et al., 2013; Quarini et al., 2016). Importantly, this association is apparent in female, but not male offspring. This female-specific sensitivity appears to be linked to prenatal conditions, as others observed that male offspring are significantly associated with high postnatal, but not prenatal, maternal depressive symptoms (Cowell et al., 2021; Myers and Johns, 2019; Quarini et al., 2016). Likewise, the association between antenatal maternal depressive symptoms and child socio-emotional problems, which predict the later risk for depression, is consistently stronger in females compared with male offsprings (see Meaney, 2018 for a systematic review).

The fetal sex-dependent association of antenatal depressive symptoms with maternal DNA methylation profiles may indicate a sex difference in the response of the fetus to the maternal condition, reflected in placental DNA methylation profiles. Indeed there is ample evidence from both rodent and human studies for placental responses to maternal stress (Bronson and Bale, 2016) as well as for sex-dependent placental responses to maternal health conditions. We used fetus-facing placenta DNA methylation from the available GUSTO placental samples to investigate to examine a sex-dependent fetal response to maternal antenatal depressive symptoms. We observed a female-specific methylation profile associated with maternal depressive symptoms (Figure 5A). We suggest that the fetal placenta of female, but not male fetuses, responds to maternal depressive symptoms and a signal that then contributes to the EPDS-associated DNA methylation pattern in maternal blood. Whereas the identity of fetal this signal remains to be determined, the Clifton findings noted above are potentially informative with respect to sex-specific placental responses to maternal health conditions. These findings implicate glucocorticoid – cytokine signaling.

An obvious issue is the degree to which DNA methylation patterns in antenatal maternal blood and fetal-facing placenta tissue might inform on the relevant mechanisms of maternal transmission. Recall that prenatal maternal depressive symptoms predict the later risk for depression in the female but not male offspring (Pearson et al., 2013). We addressed this issue by mapping unique genes from the EPDS-vCpGs derived from maternal blood methylome and fetal-facing placental methylome. Interestingly, we found that the pattern of tissue-specific expression of genes mapped from EPDS-vCpGs of mothers with female fetuses, relative to all vCpGs found in the maternal blood methylome, showed a strikingly greater enrichment in the brain (Figure 4A). Moreover, there was enrichment of these vCpGs in multiple brain regions closely associated with MDD including the frontal cortex (BA9), anterior cingulate cortex (BA24), the basal ganglia, and notably nucleus accumbens, as well as the hippocampus and amygdala (Figure 4B; Akil et al., 2018; Drysdale et al., 2017; Hamilton et al., 2013; Otte et al., 2016; Schmaal et al., 2016; Siddiqi et al., 2021). Neuroimaging studies of MDD patients compared with controls reveal significant differences in structure and connectivity for each of these regions (see Zhuo et al., 2019 for a review). Moreover, the nucleus accumbens and anterior cingulate cortex are regions targeted by deep brain stimulation as an effective treatment for severe MDD (Bewernick et al., 2010; Puigdemont et al., 2012; Schlaepfer et al., 2008). Finally, we used an online database of GWAS catalogs (FUMA; https://fuma.ctglab.nl) and found that genes associated with the maternal EPDS scores in women bearing a female fetus were significantly enriched for those associated with clinical depression (FDR *p* = 3.37 × 10^−4^). Taken together these findings suggest that the fetal sex-specific associations of maternal depressive symptoms and DNA methylation are related to the biology of MDD.

Mapping the EPDS-vCpGs of maternal blood methylome to genes also permitted informatic analysis of candidate biological pathways (Table 2). The top biological process “Positive regulation of WNT/β-catenin signaling at the receptor level” is involved in its development. WNT ligands act through frizzled receptors to activate β-catenin (MacDonald et al., 2009). The activation of this canonical WNT signaling cascade results in the stabilization of cytosolic β-catenin, its translocation to the nucleus and downstream effects on gene transcriptional. Activation of WNT/β-catenin signaling is implicated in neural development (Ciani and Salinas, 2005; Logan and Nusse, 2004), consistent with the identification of the “Development” as the primary biological process linked to maternal EPDS-vCpGs. There is also compelling evidence for the relevance of the WNT/β-catenin signaling in clinical depression (Gould et al., 2008; Li and Jope, 2010; Maguschak and Ressler, 2012). β-catenin expression is enriched in those regions showing enrichment for genes mapped from EPDS-vCpGs and functionally linked to MDD, including the prefrontal cortex, nucleus accumbens, amygdala, hypothalamus, and hippocampus (see Teo et al., 2018). WNT/β-catenin expression is regulated by multiple classes of antidepressant medications (Okamoto et al., 2010) as well as electroconvulsive therapy (Madsen et al., 2003). Moreover, differential activation of WNT/β-catenin signaling mediates the depression-like behavioral effects of chronic stress in pre-clinical models of depression (e.g., Dias et al., 2014; Wilkinson et al., 2011). In sum, analysis of maternal EPDS-vCpGs in mother bearing female fetuses identifies biological pathways linked to MDD.

The results of the transcription factor enrichment are both consistent with previous studies of maternal depression and instructive with respect to potential mechanisms for chromatin remodeling. Mehta et al. (2014) previously identified 116 genes, the expression of which in the third trimester of pregnancy predicted postpartum depression scores. These transcripts were significantly enriched for estrogen receptor targets and showed dynamic changes in expression over the perinatal period in women with higher levels of postpartum depressive symptoms. This team (Mehta et al., 2019) found that DNA methylation levels across these same estrogen receptor-sensitive genes are also associated with postpartum depressive symptoms. The results suggest that perinatal depressive symptoms are linked to inter-individual variation in estrogen receptor sensitivity.

Estrogen receptor activation can directly remodel DNA methylation (Kangaspeska et al., 2008; Métivier et al., 2008). Estrogen receptor activation enhances the expression of p300/CBP, a histone acetyltransferase, which can initiate the remodeling of DNA methylation (Szyf, 2009). Additionally, the transcription factor enrichment analysis identified *GATA2*, a pioneer transcription factor, as well as both the *ESR1 and ESR2* (Tables S3 and S5). Pioneer transcription factors, such as *GATA2*, interact with sex steroid receptors to initiate chromatin remodeling (e.g., Li et al., 2021; Wall et al., 2014). These findings are consistent with the results of our analyses showing an enrichment for these factors in genes mapped from EPDS-vCpGs of both maternal blood and fetal-facing placenta methylome, apparent only in relation to female fetuses.

### Limitations of the study

Our fetal sex-specific associations of maternal methylome with antenatal maternal depressive symptoms in the Singapore GUSTO cohort were also observed in the large, UK-based ALSPAC cohort providing some measure of confidence in the reliability of the findings. The results from bioinformatic analyses provide a strikingly novel report of an instance in which the biological profile of the mother associated with a specific health condition was dependent upon the sex of the fetus. We acknowledge that one limitation of this study is the lack of replication for placenta-specific methylome association with maternal depressive symptoms. However, we note that there is ample evidence from both rodent and human studies for placental responses to maternal stress (Bronson and Bale, 2016) as well as for sex-dependent placental responses to maternal health conditions. We consider these findings to provide a compelling direction for the study of maternal–fetal interactions and for the study of the mechanisms of intergenerational transmission.

## STAR★Methods

### Key resources table

**Table:** 

REAGENT or RESOURCE	SOURCE	IDENTIFIER
**Biological samples**		

GUSTO Maternal blood methylation data (buffy coat)	GUSTO (Growing Up In Singapore Towards healthy Outcomes cohort)	GSE158063
ALSPAC Maternal blood methylation data	ALSPAC (Avon Longitudinal Study of Parents and Children cohort)	http://www.alspac.bris.ac.uk; RRID:SCR_007260
GUSTO fetal-facing placenta methylation data	GUSTO (Growing Up In Singapore Towards healthy Outcomes cohort)	GSE208529

**Critical commercial assays**		

QIASymphony DSP DNA Midi Kit	QIAGEN	Cat# 937255; RRID:SCR_008539
EZ DNA Methylation Kit	Zymo Research	Cat# D5002; RRID:SCR_008968
Infinium MethylationEPIC BeadChip (850K)	Illumina	Cat# WG-317-1003; RRID:SCR_010233
Infinium HumanMethylation450 BeadChip	Illumina	Cat# WG-314-1002; RRID:SCR_010233

**Deposited data**		

GUSTO Maternal blood methylation data (buffy coat)	GUSTO (Growing Up In Singapore Towards healthy	GSE158063
ALSPAC Maternal blood methylation data	ALSPAC (Avon Longitudinal Study of Parents and Children cohort)	http://www.alspac.bris.ac.uk; RRID:SCR_007260
GUSTO fetal-facing placenta methylation data	GUSTO (Growing Up In Singapore Towards healthy Outcomes cohort)	GSE208529

**Software and algorithms**		

*minfi* (R package)	Aryee et al., 2014	http://www.bioconductor.org/packages/release/bioc/html/minfi.html; RRID:SCR_012830
*Combat* (R package)	Johnson et al., 2007	http://biosun1.harvard.edu/complab/batch/; RRID:SCR_010974
MetaCore v21.1.70400	Clarivate Analytics	https://portal.genego.com; RRID:SCR_008125
*FUMA*	Watanabe et al., 2017	https://fuma.ctglab.nl; RRID:SCR_017521
R	R Core Team, 2020	https://www.r-project.org; RRID:SCR_001905
*ggplot2* (R package)	Wickham, 2016	https://cran.r-project.org/web/packages/ggplot2/index.html; RRID:SCR_014601
flowsorted.blood.epic (R package)	Salas et al., 2018	https://bioconductor.org/packages/FlowSorted.Blood.EPIC/
*meffil*	Min et al., 2018;	https://github.com/perishky/meffil

### Resource availability

#### Lead contact

Further information and requests for resources should be directed to and will be fulfilled by the Lead Contact, Dr Michelle Kee (michelle_kee@sics.a-star.edu.sg).

#### Materials availability

This study did not generate new unique reagents.

### Experimental model and subject details

Pregnant women at 7–11 weeks of pregnancy were prospectively recruited from two maternity hospitals in Singapore between June 2009 and October 2010, to participate in the Growing Up in Singapore Towards Healthy Outcomes (GUSTO) birth cohort study (Soh et al., 2014). These women had the following inclusion criteria: aged 18 years old and above, were Singapore Citizens or Singapore Permanent Residents, intend to deliver at either of the two maternity hospitals (National University Hospital or KKH), intend to reside in Singapore for the next 5 years, willing to donate cord, cord blood and placenta, and the fetus should be racially homogenous with both sets of grandparents of the same ethnicity. Women with significant medical conditions (e.g., Type 1 diabetes mellitus), on certain medications including psychotropic drugs, on chemotherapy, or mixed marriages were not recruited into the cohort. Women whose pregnancies end in miscarriages were excluded later as well. The GUSTO study was approved by the National Healthcare Group Domain Specific Review Board (D/09/02) and the SingHealth Centralized Institutional Review Board (2009/280/D). Informed written consents were obtained from all participants in this study.

Our analyses also included data from the Avon Longitudinal Study of Parents and Children (ALSPAC) cohort, a population-based cohort from the Avon county in the United Kingdom (Boyd et al., 2013; Fraser et al., 2013; Northstone et al., 2019). Pregnant women in the ALSPAC cohort were recruited if they were Avon residents while pregnant, and their expected delivery dates lie between 1st April 1991 and 31st December 1992 (N = 14,541). Additional recruitment (N = 913) was done during later phases, bringing the total sample size to 15,454 pregnancies, resulting in 15,589 foetuses. Of these 14,901 were alive at 1 year of age. Consent for biological samples had been collected following the Human Tissue Act (2004). Ethical approval for the ALSPAC data collection was obtained from the ALSPAC Law and Ethics Committee and the Local Research Ethics Committees (a full list of the ethics committees that approved different aspects of the ALSPAC studies is available at http://www.bristol.ac.uk/alspac/researchers/research-ethics/). Data were collected during clinic visits or with postal questionnaires. Please note that the study website contains details of all the data that is available through a fully searchable data dictionary and variable search tool at http://www.bristol.ac.uk/alspac/researchers/our-data/. Informed consent for the use of data collected via questionnaires and clinics was obtained from participants following the recommendations of the ALSPAC Ethics and Law Committee at the time.

Participants who matched the following criteria were included in our analyses (Figure 1): either Chinese from the GUSTO cohort (*n* = 491) or Caucasians (*n* = 387) from the ALSPAC cohort, aged 18 years old and above, singleton births, had both methylation and prenatal EPDS data, child’s gestational age of 37–42 weeks inclusively, and child’s birth weight of ≥ 2kg. A summary of the demographics of these women can be found in Table 1.

### Method details

#### Measures

For both the GUSTO and ALSPAC cohort, maternal demographics and social economic status such as maternal age, marital status, mothers’ education, and household income were collected during clinic visits or with postal questionnaires. Child’s sex and birth weight were obtained from the hospital medical records. Gestational age of the fetus for the GUSTO cohort was estimated using ultrasonography in first trimester of pregnancy.

Depressive symptoms were assessed using the Edinburgh Postnatal Depression Scale (EPDS; Cox et al., 1987) during 26^th^ – 28^th^ weeks of pregnancy in the GUSTO cohort and at 32^nd^ week of pregnancy in the ALSPAC cohort respectively. The EPDS is a validated self-report instrument that contains 10 items of common depressive symptoms over the past week, and is both sensitive and reliable in detecting prenatal depression in women (Matthey et al., 2006).

#### Biosample collection and processing

Blood from GUSTO mothers (≤10 mL) directly into EDTA tubes during their 26^th^ – 28^th^ weeks of pregnancy. Blood samples were then centrifuged at 4°C, at 1600g for 10 min to separate into three distinct layers – plasma, buffy coat and erythrocytes. The middle buffy coat layer was extracted carefully and stored at −80°C before purification. QIASymphony SP was then used for automated purification of buffy coat DNA in combination with QIASymphony DSP DNA Midi Kit (Qiagen, Cat #: 937255) as per manufacturer’s instructions. Genomic DNA was bisulfite converted using the Zymo Research’s EZ DNA Methylation Kit (Cat #: D5002). Peripheral blood samples from ALSPAC mothers were collected according to standard procedures, as previously described (Relton et al., 2015).

Placenta tissues from a subset of the GUSTO cohort were collected immediately upon delivery. These placenta tissues were previously selected for further analyses on long term effects following conception via *in vitro* fertilization (IVF). Hence, these subjects with placenta collected were further categorized into 3 groups: high risk of infertility, IVF and normal population. Five sections of the placenta facing the fetal side were cut ∼2 cm away from the site of cord insertion roughly equidistant from one another. The sections were then rinsed with phosphate buffer saline (PBS) solution and cut into smaller pieces, before snap-freezing with liquid nitrogen and stored at −80°C in 2-mL cryovials before DNA extraction. Frozen sections of placenta tissues were crushed with a new aluminium-lined mortar and pestle in liquid nitrogen. The mortar and pestle were also washed with 70% ethanol between each sample. 75 mg (+/− 10%) of crushed placenta tissue and added 500 μL Buffer ATL (Qiagen, Cat #: 939011) and 30 μL Proteinase K (from QIASymphony DSP DNA Midi Kit) and incubated overnight in a shaking incubator. Thereafter, the steps were similar to DNA extraction from maternal blood of the GUSTO cohort. Briefly, QIASymphony SP was then used for automated purification of placenta DNA in combination with QIASymphony DSP DNA Midi Kit as per manufacturer’s instructions.

#### DNA methylation profiling

DNA methylation profiling was performed on maternal buffy coat and fetal-facing placenta tissues, using Infinium MethylationEPIC BeadChip (850K; “EPIC 850K”) for the GUSTO cohort. For the ALSPAC cohort, DNA methylation profiling was performed on maternal buffy coat cells and the Infinium HumanMethylation450 BeadChip (“Infinium 450K”). Quality control and pre-processing steps of raw DNA methylation .idat files from both cohorts were done in R using the *minfi* package (Aryee et al., 2014). Briefly, probes with fewer than three beads for either the methylated or unmethylated channel, or with detection p ≥ 0.01 were removed. Probes containing SNPs at the CpG site or its single-base extension and cross-hybridizing probes (McCartney et al., 2016) were also removed. Within-sample normalization was performed using *Noob* pre-processing (Triche et al., 2013). The beta values were first converted to M-value to remove chip effect observed in the data using *ComBat* (Johnson et al., 2007). The M-values adjusted for chip were then converted back to beta values for subsequent analysis. Finally, we filter for variable CpGs (“vCpGs”) by excluding probes where the methylation range (maximum-minimum, excluding outliers) less than 5%.

For the ALSPAC cohort, DNA methylation profiling was performed on maternal buffy coat samples (https://alspac.github.io/omics_documentation/methylation/user_guide_ARIESreleasev3.docx) and the Infinium HumanMethylation450 BeadChip (“Infinium 450K”). Quality control and pre-processing steps of raw DNA methylation was carried out by ALSPAC with *meffil* (Min et al., 2018; https://github.com/perishky/meffill). Similar to GUSTO, probes with less than three beads or with detection p ≥ 0.01 were removed. Samples were excluded if they failed genotyping QC (genotype concordance less than 80% between 65 SNPs probes on the array and external genotypes, sample swaps, gender mismatches, high identity by descent (IBD) or relatedness issues), were methylated versus unmethylated outliers, had dye bias issues. Functional normalization procedure (*meffil*) was applied to methylated and unmethylated intensities separately, and to type I and type II signals separately (the slide was regressed out on the raw betas before normalization). Following the normalization procedure, we excluded technical duplicates (the sample with the highest number of detected probes was kept). Same as in GUSTO, only vCpGs (maximum-minimum, excluding outliers greater or equal to 5%) were considered for the analysis.

The final number of vCpGs from maternal blood used in this study were 422,691 and 410,353 vCpGs for the GUSTO and ALSPAC cohorts respectively. These vCpGs are from autosomal chromosomes and chromosome X. 187,935 vCpGs were commonly found in both the GUSTO and ALSPAC cohorts. A final number of 580,442 vCpGs from autosomal chromosomes of fetal-facing placenta tissue were used in this study from the GUSTO cohort.

### Quantification and statistical analysis

All statistical analyses were performed in R (R Core Team, 2020). Linear regression analysis in the ALSPAC cohort was performed on beta values against EPDS scores, adjusting for the first five principal components of estimated cellular composition (Ecc). Similarly, linear regression analysis in the GUSTO cohort included the first five principal components of estimated cellular compositions as well as technical covariates (ie: chip position, DNA extraction method and bisulfite-converted DNA concentration level). Cellular proportions in GUSTO were estimated using an adult blood reference panel in FlowSorted.Blood.EPIC R package (Salas et al., 2018) and in ALSPAC using Houseman et al. (2012) method. Specifically, the linear regression equation for each cohort is:

ALSPAC (maternal blood): beta ∼ EPDS scores + EccPCs

GUSTO (maternal blood): beta ∼ EPDS scores + EccPCs + Chip Position + DNA extraction method + bisulfite-converted DNA concentration level

GUSTO (placenta): beta ∼ EPDS scores + EccPCs + Chip Position + groups of mothers

Smoking during pregnancy was not added as a covariate as it was not associated with the top 5 PCs of methylation data in both the GUSTO and ALSPAC cohorts.

Separate linear regression analyses were performed for mothers bearing male and female babies. One-tailed Kolmogorov-Smirnov test was done to test the uniformity of vCpGs associated with EPDS scores for both the GUSTO and ALSPAC cohort.

#### Biological pathway, transcription factor and tissue specificity analyses

The 2417 and 3619 unique genes mapped from the maternal EPDS-vCpGs bearing female fetus and EPDS-vCpGs from female fetus-facing placenta tissues were respectively imported into MetaCore^TM^ (v21.1.70400; Clarivate Analytics) for pathway enrichment and transcription factor analyses. 25,641 and 25, 281 unique genes mapped from the vCpGs of both maternal blood and placenta sources were used respectively as the reference list. *P*-values for transcription factor analyses were determined using hypergeometric intersection.

Each input gene mapped from maternal EPDS-vCpGs bearing female fetuses was also queried against previously associated phenotypes using FUMA (Watanabe et al., 2017). FUMA was also used to examine the up-regulated expression of these genes mapped from EPDS-vCpGs, relative to genes mapped from vCpGs, in specific tissues based on GTex v8 RNA-seq data (GTEx Consortium and Ardlie, 2015). Bonferroni corrected *p*-values were provided for the up-regulated differentially expressed gene sets from FUMA.

### Additional resources

The GUSTO cohort is registered under ClinicalTrials.gov as NCT01174875.

## Data Availability

The GUSTO methylation data reported in this study is a subset of GSE158063 and GSE208529. Any additional information required to reanalyze the data reported in this paper is available from the lead contact or Mr P Mukkesh Kumar (Mukkesh_Kumar@sics.a-star.edu.sg) upon request. To request access, please contact Dr Michelle Kee or Mr P Mukkesh Kumar to submit additional information before data access is given. This paper does not report original code.
