# Supplementary material for: Fetal sex-specific epigenetic associations with prenatal maternal depressive symptoms
Source: iScience. 2022 Aug 4;25(9):104860. doi: 10.1016/j.isci.2022.104860 (PMC9421382; doi:10.1016/j.isci.2022.104860)
Supplement: Document S1. Figures S1–S3 and Tables S1–S5 [file mmc1.pdf]

iScience, Volume *25*

Supplemental information

Fetal sex-specific epigenetic associations with prenatal maternal depressive symptoms

Michelle Z.L. Kee, Ai Ling Teh, Andrew Clappison, Irina Pokhvisneva, Julie L. MacIssac, David T.S. Lin, Katia E. Ramadori, Birit F.P. Broekman, Helen Chen, Mary Lourdes Daniel, Neerja Karnani, Michael S. Kobor, Peter D. Gluckman, Yap Seng Chong, Jonathan Y. Huang, and Michael J. Meaney

Supplementary Figures Legends

Figure S1. *P*-values distribution of non-vCpGs from GUSTO maternal methylome associated with prenatal EPDS scores for mothers carrying either female babies (A) or male babies (B), Related to Figure 1. Dashed lines represent the uniform distribution that was expected by chance. Non-vCpGs from the maternal blood methylome of the GUSTO cohort showed no association with prenatal maternal depressive symptoms (KS test $p = 0.37$ and > 0.99 for females and males respectively.

Figure S2*. P*-values distribution of maternal methylation associated with prenatal EPDS scores for mothers carrying either female babies (A,C) or male babies (B,D) at a threshold of $p < 0.001$, Related to Figure 1. Dashed lines represent the uniform distribution that was expected by chance. Top panels refer to data from GUSTO mothers, while bottom panels refer to data from ALSPAC mothers.

Figure S3. *P*-values distribution of non-vCpGs from GUSTO fetal-facing placental methylome associated with prenatal EPDS scores for mothers carrying either female (A) or male babies (B), Related to Figure 5. Dashed lines represent the uniform distribution that was expected by chance. Non-vCpGs from the fetal-facing placental methylome of the GUSTO cohort showed no association with prenatal maternal depressive symptoms (KS test $p > 0.99$ for both females and males).

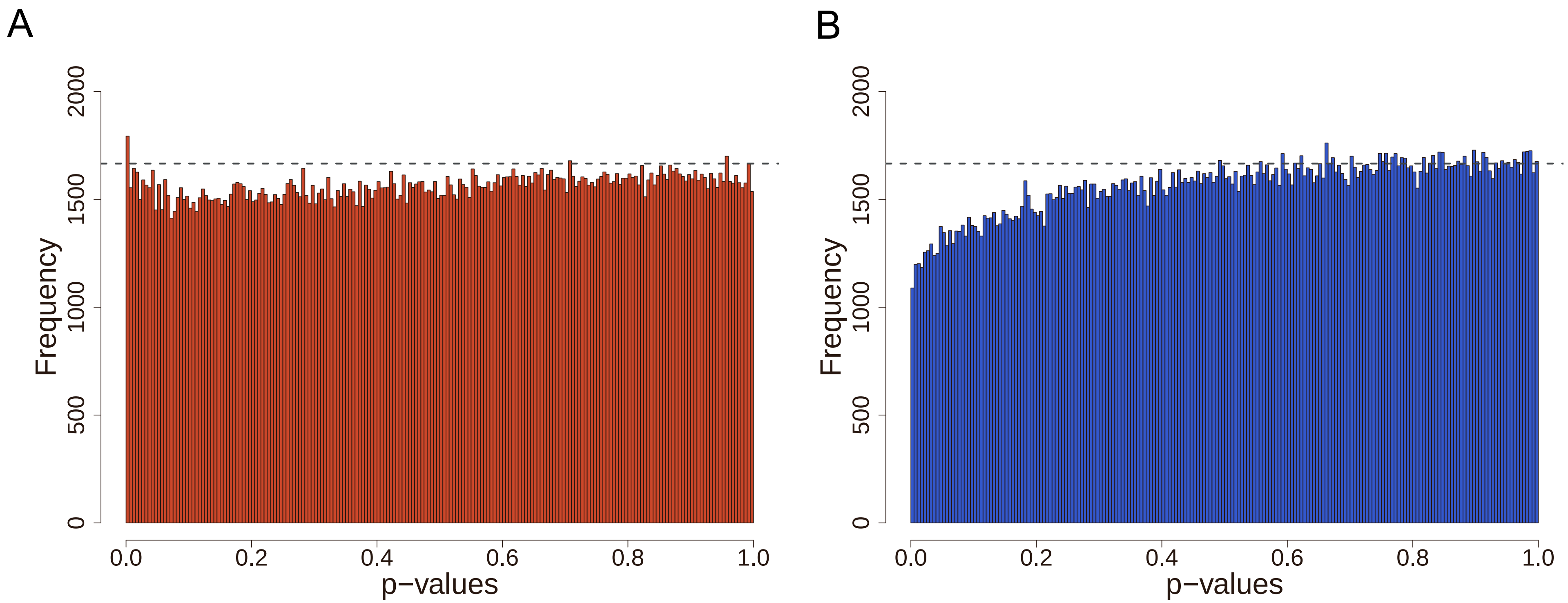

Figure S1. P-values distribution of non-vCpGs from GUSTO maternal methylome associated with prenatal EPDS scores for mothers carrying either female (A) or male babies (B), Related to Figure 1.

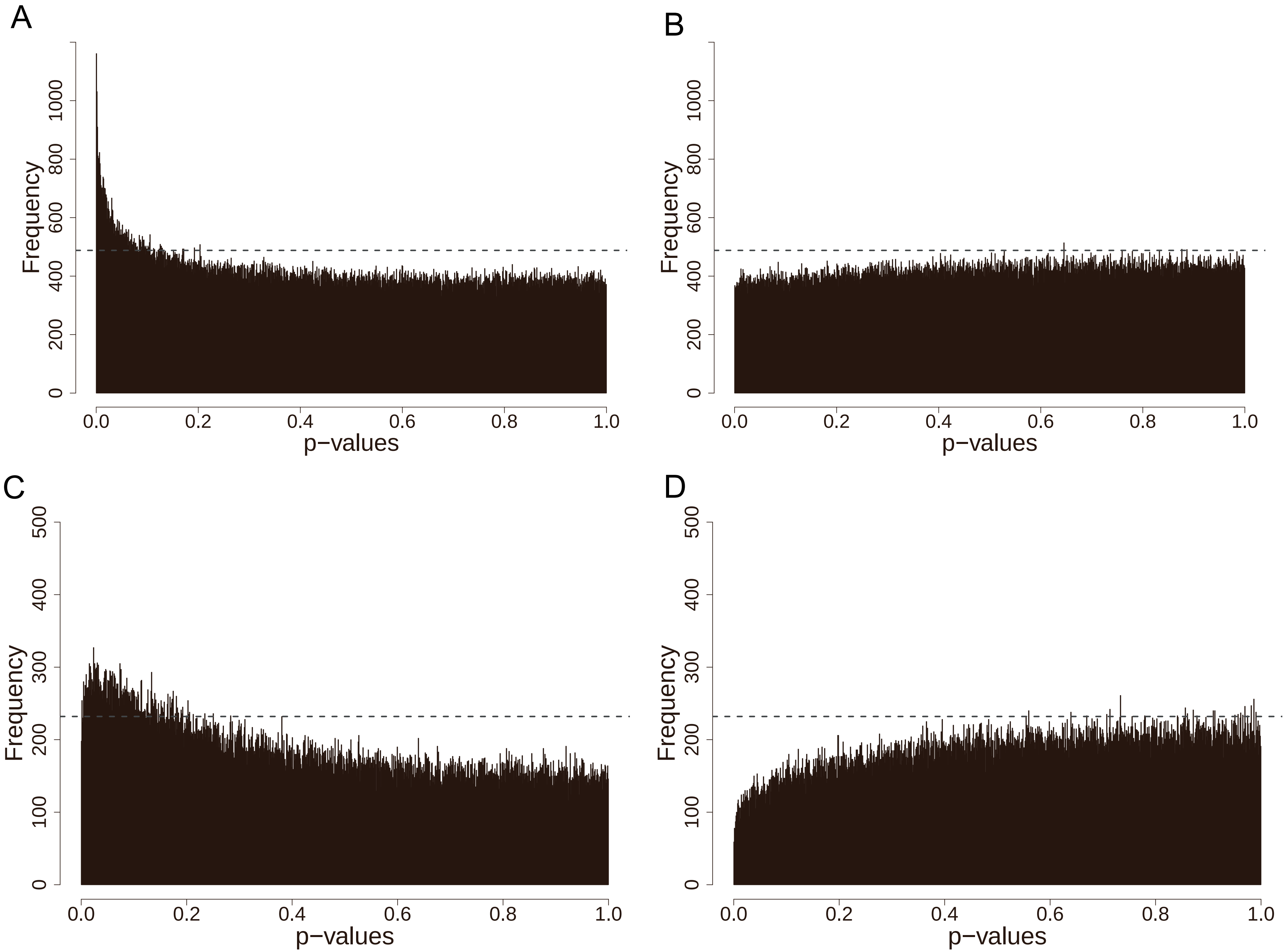

Figure S2. P-values distribution of maternal methylation associated with prenatal EPDS scores for mothers carrying either female (A, C) or male (B,D) babies at a threshold of $p < 0.001$, Related to Figure 1.

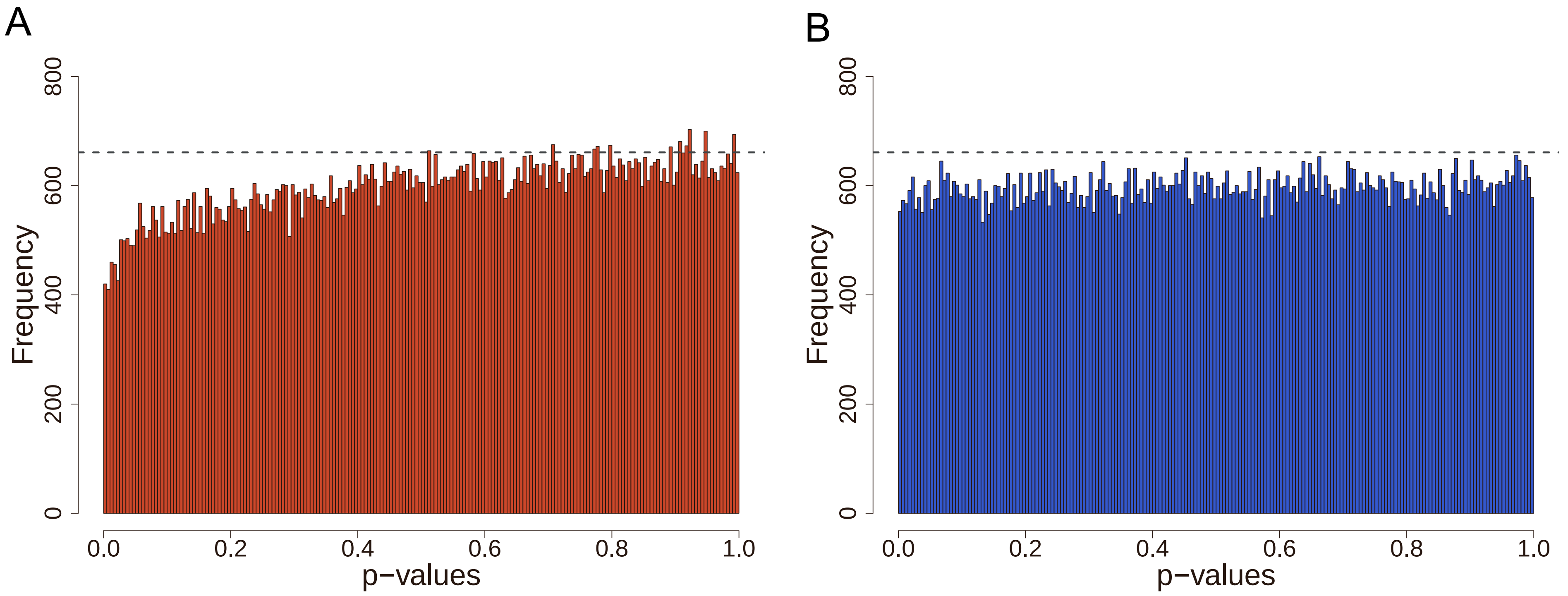

Figure S3. P-values distribution of non-vCpGs from GUSTO fetal-facing placental methylome associated with prenatal EPDS scores for mothers carrying either female (A) or male babies (B), Related to Figure 5.

Table S1. Genomic region distributions of vCpGs relative to total CpGs in EPIC 850K, and EPDS-vCpGs relative to vCpGs, Related to Figure 4.

Regions	850K	vCpGs	EPDS-vCpGs (male)	EPDS-vCpGs (female)	Enrichment			Depletion		
					p	*p* (male)	*p* (female)	*p*	*p* (male)	*p* (female)
Island	161441	39808	332	191	1	4.28E-31	1	<2.22E-308	1	1.48E-45
Shore	154546	79509	456	651	1.88E-115	4.07E-11	1	1	1	2.49E-20
Shelf	61691	32365	128	356	4.56E-79	.86	.62	1	.16	.40
Open sea	488181	271009	909	3518	<2.22E-308	1	2.31E-54	1	4.65E-36	1
Promoter	200248	70408	345	556	1	2.20E-13	1	<2.22E-308	1	3.96E-16
5'UTR	10482	3708	21	26	1	1.68E-02	.99	7.40E-172	.99	1.44E-02
Exon	66625	28810	103	242	1	.28	1	3.04E-198	.75	4.06E-05
Intron	328054	173672	512	1820	<2.22E-308	1	.81	1	3.38E-05	.20
3'UTR	17734	8775	29	82	3.76E-02	.57	.90	.96	.50	.12
TTS	16937	8500	34	55	1.65E-04	.18	1	1	.86	3.20E-05
Intergenic	225779	128818	382	1718	<2.22E-308	1	1.04E-28	1	1.21E-03	1

TTS refers to transcription termination sites.

Table S2. Top 30 biological pathways of genes mapped from EPDS-vCpGs of mothers with female fetuses, Related to Table 2.

No.	Biological pathways		*p*	FDR
1	Development	Positive regulation of WNT/Beta-catenin signaling at the receptor level	4.73E-11	6.80E-08
2	Main genetic and epigenetic alterations in lung cancer		1.26E-08	9.10E-06
3	Signal transduction	Adenosine A1 receptor signaling pathway	3.88E-08	1.78E-05
4	Putative role of Estrogen receptor and Androgen receptor signaling in progression of lung cancer		6.41E-08	1.78E-05
5	Nociception	Nociceptin receptor signaling	7.42E-08	1.78E-05
6	Gamma-secretase proteolytic targets		7.42E-08	1.78E-05
7	Signal transduction	Cyclic AMP signaling	9.35E-08	1.92E-05
8	Development	Thromboxane A2 signaling pathway	1.71E-07	3.08E-05
9	Signal transduction	PKA signaling	2.38E-07	3.80E-05
10	Transcription targets of Androgen receptor involved in Prostate Cancer		4.39E-07	6.32E-05
11	Epigenetic alterations in ovarian cancer		1.38E-06	1.70E-04
12	Gamma-Secretase regulation of neuronal cell development and function		1.41E-06	1.70E-04
13	Signal transduction	Intracellular calcium increase	1.67E-06	1.85E-04
14	Signal transduction	WNT/Beta-catenin signaling in tissue homeostasis	2.76E-06	2.84E-04
15	Signal transduction	Angiotensin II/ AGTR1 signaling via p38, ERK and PI3K	3.31E-06	3.18E-04
16	Development	WNT/Beta-catenin signaling in embryogenesis	3.79E-06	3.21E-04
17	Signal transduction	Modulation of calcium and potassium channels by Adenosine A1 receptor	3.79E-06	3.21E-04
18	Tinnitus-associated changes in auditory pathway		5.53E-06	4.43E-04
19	Cell cycle progression in Prostate Cancer		6.27E-06	4.62E-04
20	G protein-coupled receptors signaling in lung cancer		6.41E-06	4.62E-04
21	WNT signaling in HCC		8.61E-06	5.90E-04
22	Protein folding and maturation	Insulin processing	1.03E-05	6.74E-04
23	Breast cancer (general schema)		1.17E-05	7.32E-04
24	WNT signaling in gastric cancer		1.43E-05	8.34E-04
25	Neurophysiological process	Dynein-dynactin motor complex in axonal transport in neurons	1.53E-05	8.34E-04
26	Development	Dopamine-induced transactivation of EGFR in SVZ neural stem cells	1.66E-05	8.34E-04

27	Gamma-secretase regulation of mammary cell development		1.67E-05	8.34E-04
28	Signal transduction	HTR2A signaling outside the nervous system	1.68E-05	8.34E-04
29	Neurophysiological process	Regulation of intrinsic membrane properties and excitability of cortical pyramidal neurons	1.68E-05	8.34E-04
30	Androgen receptor activation and downstream signaling in Prostate cancer		1.78E-05	8.55E-04

Table S3. Top 30 transcription factors that regulate genes mapped from EPDS-vCpGs of mothers carrying female fetuses, Related to Table 2.

Gene ID	Transcription Factors	z-score	*p*	FDR
**ESRRG**	Esrrg	30.41	0	0
**GATA2**	GATA-2	32.19	0	0
**TCF7L2**	TCF7L2 (TCF4)	25.16	9.74E-97	3.31E-95
**ASCL2**	ASH2	20.28	4.20E-69	1.07E-67
**HEBP1**	NRF2	17.47	4.02E-51	8.19E-50
**ESR1**	ESR1 (nuclear)	16.32	4.99E-46	8.48E-45
**AR**	Androgen receptor	13.5	1.79E-32	2.60E-31
**MEIS1**	MEIS1	13.56	2.90E-32	3.69E-31
**HNF4A**	HNF4-alpha	11.03	3.82E-24	4.33E-23
**VDR**	VDR	11.88	1.16E-23	1.18E-22
**TCF7**	TCF7 (TCF1)	10.63	2.15E-22	1.99E-21
**HIF1A**	HIF1A	9.347	9.53E-17	8.10E-16
**ESR2**	ESR2 (nuclear)	9.403	7.19E-16	5.64E-15
**TFCP2L1**	LBP9	8.464	9.03E-15	6.58E-14
**RBP3**	E2F1	7.836	4.16E-14	2.83E-13
**HNF4A**	HNF1-alpha	8.743	6.95E-13	4.43E-12
**CUX1**	CUX1 (p110)	7.662	1.98E-12	1.19E-11
**NFATC1**	NF-AT2(NFATC1)	7.635	1.85E-10	1.05E-09
**WT1**	WT1	7.351	5.22E-10	2.80E-09
**LIM2**	LHX2	7.172	5.37E-10	2.74E-09
**NR5A2**	LRH1	7.1	8.19E-09	3.98E-08
**KLF5**	KLF5	6.514	4.99E-08	2.31E-07
**PAX8**	PAX8	6.591	5.13E-08	2.28E-07
**SMAD2**	SMAD2	5.607	1.47E-06	6.23E-06
**PAX5**	PAX5	5.491	2.03E-06	8.29E-06
**PAX6**	PAX6	5.199	3.12E-06	1.22E-05
**MLXIPL**	ChREBP	5.493	4.66E-06	1.76E-05
**RORC**	ROR-gamma	5.394	4.74E-06	1.73E-05
**NR4A1**	NUR77	4.908	3.90E-05	1.37E-04
**FOXP2**	FOXP2	4.528	4.55E-05	1.55E-04

Table S4. Top 30 biological pathways of genes mapped from EPDS-vCpGs of female fetus placenta tissue (fetal-side facing), Related to Table 3.

No.	Biological pathways		*p*	FDR
1	Development	NCAM1-mediated neurite outgrowth, synapse assembly and neuronal survival	1.11E-06	1.656E-03
2	G-protein signaling	Rac1 activation	3.53E-06	2.295E-03
3	Neurophysiological process	ACM1, ACM3 and ACM5 signaling in the brain	4.63E-06	2.295E-03
4	Development	PIP3 signaling in cardiac myocytes	7.74E-06	2.877E-03
5	Protein folding and maturation	Insulin processing	1.26E-05	3.735E-03
6	Chemotaxis	SDF-1/ CXCR4-induced chemotaxis of immune cells	1.65E-05	4.090E-03
7	Neurophysiological process	Netrin-1 in regulation of axon guidance	3.58E-05	7.612E-03
8	Cytoskeleton remodeling	Regulation of actin cytoskeleton nucleation and polymerization by Rho GTPases	4.55E-05	8.028E-03
9	Immune response	Immunological synapse formation	5.08E-05	8.028E-03
10	Signal transduction	Cyclic AMP signaling	5.40E-05	8.028E-03
11	G-protein signaling	Rap1A regulation pathway	7.97E-05	1.077E-02
12	Transcription	CREB signaling pathway	1.07E-04	1.305E-02
13	Regulation of metabolism	GLP-1-induced insulin secretion	1.14E-04	1.305E-02
14	Development	ROBO2, ROBO3 and ROBO4 signaling pathways	1.42E-04	1.504E-02
15	Development	Role of HDAC and calcium/calmodulin-dependent kinase (CaMK) in control of skeletal myogenesis	1.70E-04	1.683E-02
16	GLP-1 in inhibition of insulin secretion in type 2 diabetes		2.11E-04	1.961E-02
17	Immune response	Platelet activating factor/ PTAFR pathway signaling	2.50E-04	2.166E-02
18	Immune response	TCR alpha/beta signaling pathway	2.62E-04	2.166E-02
19	Muscle contraction	Regulation of eNOS activity in cardiomyocytes	2.97E-04	2.326E-02
20	Immune response	NF-AT in immune response	3.64E-04	2.504E-02
21	Cytoskeleton remodeling	FAK signaling	3.66E-04	2.504E-02
22	Immune response	Histamine signaling in dendritic cells	3.70E-04	2.504E-02
23	Cytoskeleton remodeling	PDGF signaling via calcium and Rho GTPases	5.10E-04	3.080E-02
24	Cytoskeleton remodeling	Role of PKA in cytoskeleton reorganisation	5.22E-04	3.080E-02
25	Neurophysiological process	Regulation of intrinsic membrane properties and excitability of cortical pyramidal neurons	5.24E-04	3.080E-02
26	Regulation of lipid metabolism	Insulin regulation of fatty acid metabolism	5.38E-04	3.080E-02
27	Immune response	IL-3 signaling via ERK and PI3K	5.70E-04	3.138E-02

28	Apoptosis and survival	NGF/ TrkA PI3K-mediated signaling	6.23E-04	3.311E-02
29	Transport	Induction of Macropinocytosis	6.76E-04	3.465E-02
30	Development	Fetal brown fat cell differentiation	7.38E-04	3.654E-02

Table S5. Top 30 transcription factors that regulate genes mapped from EPDS-vCpGs in female fetus placenta tissue (fetal-side facing), Related to Table 3.

Gene ID	Transcription Factors	z-score	*p*	FDR
**TFCP2L1**	LBP9	23.76	6.31E-128	1.04E-125
**RUNX1**	AML1 (RUNX1)	17.53	6.33E-66	5.19E-64
**TCF7L2**	TCF7L2 (TCF4)	16.84	2.19E-58	1.20E-56
**ETS1**	ETS1	16.37	2.94E-58	1.21E-56
**C11orf9**	C11orf9	14.8	7.44E-43	2.44E-41
**SMAD1**	SMAD1	13.77	1.55E-41	4.24E-40
**RBP3**	E2F1	12.7	4.31E-35	1.01E-33
**SPI1**	PU.1	11.32	2.47E-26	5.06E-25
**GATA2**	GATA-2	10.41	2.59E-25	4.72E-24
**POU5F1**	Oct-3/4	10.12	3.34E-23	5.47E-22
**ESR1**	ESR1 (nuclear)	9.712	1.23E-20	1.83E-19
**TCF7**	TCF7 (TCF1)	9.251	4.19E-19	5.73E-18
**NFATC2**	NF-AT1 (NFATC2)	8.313	8.75E-16	1.10E-14
**TCF7L1**	TCF7L1 (TCF3)	7.543	1.61E-13	1.89E-12
**ESR2**	ESR2 (nuclear)	7.676	3.28E-13	3.59E-12
**SRA1**	SOX9	6.76	5.08E-11	5.21E-10
**TEAD1**	TEF-1	6.048	1.78E-09	1.72E-08
**RELA**	RelA (p65 NF-kB subunit)	5.675	1.99E-08	1.82E-07
**PBX2**	PBX2	5.769	4.11E-08	3.55E-07
**TCF3**	E2A	5.584	5.71E-08	4.68E-07
**PPARA**	PPAR-alpha	5.678	6.57E-08	5.13E-07
**MEIS2**	MEIS2	5.524	1.53E-07	1.14E-06
**MPP2**	FOXM1	5.163	4.56E-07	3.25E-06
**NR1H4**	FXR	5.168	7.76E-07	5.30E-06
**HSF1**	HSF1	5.099	1.01E-06	6.61E-06
**ERG**	ERG	4.882	2.37E-06	1.49E-05
**IRF1**	IRF1	4.534	5.61E-06	3.41E-05
**KLF15**	KLF15	4.96	1.41E-05	8.26E-05
**LEF1**	Lef-1	4.255	2.75E-05	1.55E-04
**NFE2**	NF-E2 (45 kDa)	4.461	3.64E-05	1.99E-04
